# Aging and immunotherapies: New horizons for the golden ages

**DOI:** 10.1002/aac2.12014

**Published:** 2020-09-30

**Authors:** Jamie A.G. Hamilton, Curtis J. Henry

**Affiliations:** 1 Department of Pediatrics, Emory University School of Medicine, Atlanta, Georgia, USA; 2 Aflac Cancer and Blood Disorders Center, Children’s Healthcare of Atlanta, Atlanta, Georgia, USA

**Keywords:** aging, anti-inflammatory drugs, clinical trials, immunity, immunotherapies, preclinical models

## Abstract

The life expectancy of the world’s elderly population (65 and older) continues to reach new milestones with older individuals currently comprising greater than 8.5% (617 million) of the world’s population. This percentage is predicted to approach 20% of the world’s population by 2050 (representing 1.6 billion people). Despite this amazing feat, many healthcare systems are not equipped to handle the multitude of diseases that commonly manifest with age, including most types of cancers. As the world’s aging population grows, cancer treatments continue to evolve. Immunotherapies are a new drug class that has revolutionized our ability to treat previously intractable cancers; however, their efficacy in patients with compromised immune systems remains unclear. In this review, we will discuss how aging-associated losses in immune homeostasis impact the efficacy and safety of immunotherapy treatment in preclinical models of aging. We will also discuss how these findings translate to elderly patients receiving immunotherapy treatment for refractory and relapsed cancers, as well as, strategies that could be explored to improve the efficacy of immunotherapies in aged patients.

## 1 | INTRODUCTION

### 1.1 | Aging and cancer

The world’s elderly population, those older than 65, is projected to grow to 1.6 billion individuals in the next three decades.^1^ By 2035, a “silver tsunami” is expected to occur in the United States, with the number of Americans 65 and older predicted to surpass those 18 and younger.^2^ This exceptional feat is in part attributed to the development of vaccines and antibiotics, which has increased the number of children surviving deadly childhood diseases such polio, smallpox, measles, and pneumonia,^3,4^ converging with declining birth rates. Other modern advances, such as a better understanding of nutrition, the implementation of routine exercise, more stringent clean water standards, and improvements in sewage treatment have also significantly contributed to extending the global life expectancy.^5,6^

Society greatly benefits from the presence of elderly people and we would all like to reach our golden years. However, the increased prevalence of older people places an enormous burden on global healthcare systems, in part, due to our inability to effectively treat elderly patients with various aging-associated malignancies.^2,7^ Aging and cancer are closely intertwined with 60% of new cancer diagnoses being made in adults aged 65 and older, and 70% of cancer deaths occurring in this population. Therefore, age has been identified as a major risk factor for developing cancer.^8^

The failure to achieve durable responses in elderly patients is multifactorial, and one of the barriers to success is the lack of aging-related research and clinical trials that include aged patients.^9,10^ For instance, between 2007 and 2018, an average of 40% of patients 65 and older were enrolled in clinical trials, despite constituting 60% of all patients with cancer.^11,12^ Furthermore, efficacy and safety information was only reported in 46% of these studies.^11^ The inconsistent recruitment of elderly patients in clinical trials has led to the development of treatments mainly in younger, healthier patients who typically have different biological and physiological responses.^11^

In addition to poor enrollment in clinical trials, the lack of preclinical studies conducted in aged animal models have further hampered the development of efficacious and safe drugs for aged patients.^13,14^ In the limited studies that have been conducted using aged mice and humans, it is consistently documented that aging alters the pharmacodynamics and pharmacokinetics of chemotherapies.^15,16^ A common manifestation of altered drug metabolism in aged patients is increased drug-induced toxicity, which often limits the dosage of chemotherapies that can be safely administered to elderly patients.^17–19^ Due to the reduced efficacy of chemotherapies in aged patients, there is increased interest in identifying novel therapies that can be used to effectively treat aging-associated cancers without accompanying toxicities.

Cellular and antibody-based immune-based therapies (immunotherapies) have revolutionized our ability to successfully treat patients with intractable diseases such as melanoma, non-small cell lung cancer (NSCLC), and relapsed/refractory B-cell malignancies.^20–23^ Unfortunately, the efficacy of immunotherapies has not been thoroughly investigated in patients with compromised immune systems. Given the extensive immunological decline associated with aging and the associated onset of chronic inflammation (inflamm-aging),^24,25^ in this review we discuss how aging-associated immunosenescence impacts the efficacy of immunotherapies. We will also discuss potential treatment strategies that could be combined with immunotherapies to optimize responses and mitigate adverse events (AEs) in this rapidly growing patient demographic.

## 2 | THE IMPACT OF AGING ON IMMUNITY

### 2.1 | Immunity and aging

**FIGURE 1:**
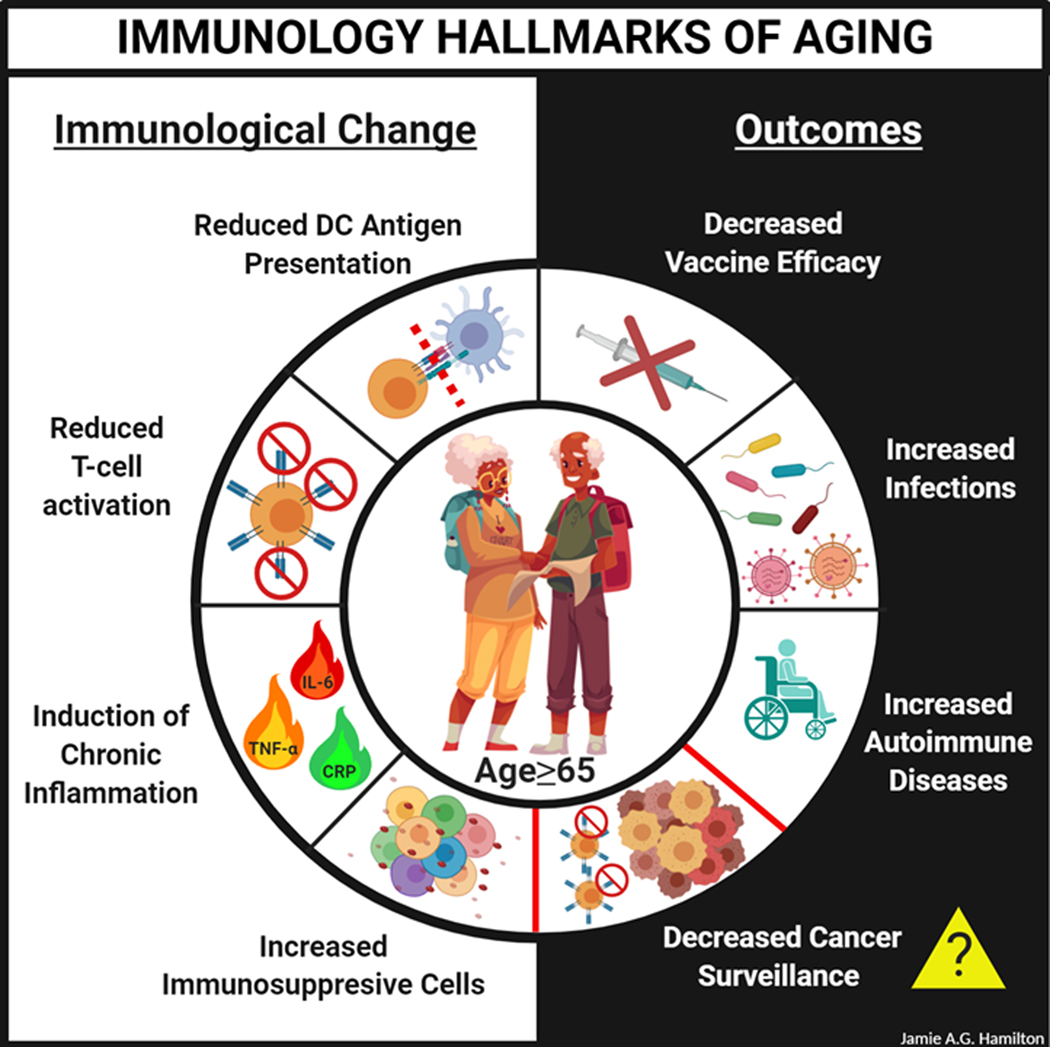
Aging is associated with a loss of immune homeostasis that contributes to various aging-associated pathologies

One hallmark of aging is immunological decline, known as immunosenescence, which has been documented in most vertebrates including mice and humans (Figure 1). This change in the immunological landscape is pleiotropic, marked by attenuated immune responses in T cells (CD4^+^ T-helper cells and CD8^+^ cytotoxic T cells), B cells, and innate immune cells such as dendritic cells (DCs), macrophages, and neutrophils.^24^ The decline in immunological integrity in elderly individuals increases their risk of infection, attenuates vaccine responses, and compromises tumor surveillance mechanisms, which increases their chances of developing cancer.^24,26–28^

#### 2.1.1 | Drivers of immunosenescence

The underlying causes of aging-associated immunosenescence are still under investigation with aging-associated chronic inflammation, known as inflamm-aging, postulated to be a major driver of compromised immunity in aged mice and humans.^29^ The onset of this state in both species is characterized by elevated circulating levels of pro-inflammatory cytokines such as IL-1*β*, IL-6, IL-8, CRP, IFN-*γ*, and TNF-*α*^29^ and shows a strong association with aging-associated multimorbidities (co-occurring diseases) including frailty,^30^ heart disease,^31^ and cognitive impairment.^32^ Emerging studies in mice have demonstrated a causal role of deregulated inflammation in perturbing immunity by altering homeostasis of hematopoietic cells.^33^ Aging in mice is characterized by a loss of stemness in hematopoietic stem cells (HSCs) and a shift toward myelopoiesis (the production of myeloid cells).^34^ The latter is due, in part, to a population shift toward myeloid-biased HSCs and fewer lymphoid-biased HSCs contributing to hematopoiesis^34^; however, emerging studies highlighting the role of inflammation are challenging this model.^35^ Pro-inflammatory cytokines such as IFN-*α*, IFN-*β*, IFN-*γ*, TGF-*β*, and TNF-*α* act directly on HSCs to regulate the production of hematopoietic progenitor cells.^36–44^ A major driver of myelopoiesis is TNF-*α*,^43,44^ which is commonly found at higher systemic levels in aged mice and humans.^45,46^ Recently, it has been demonstrated that reducing TNF-*α*, IL-1*β*, and IL-6 levels in aged mice using the anti-inflammatory mediators alpha-1-antitrypsin (AAT) and interleukin-37 (IL-37) restores fitness parameters (e.g., key mediators of cell replication) of B-progenitor cells to almost youthful levels, whereas the aging-associated decline in the numbers of B-progenitor cells was not restored.^47^ Indeed, reducing inflammation in aged mice increased IL-7 signaling and the expression of nucleotide synthesis genes in B-progenitor cells to near youthful levels.^47^ Aging-associated alterations in hematopoiesis have also been documented in humans.^33,34,48,49^ When cord blood and adult bone marrow samples from young and old individuals is compared, the frequency of immunophenotypically defined hematopoietic stem and progenitor cells (HSPCs) was reduced in aged donors and gene expression analyses of these cells revealed a myeloid-megakaryocyte-erythroid bias and reduced expression of genes involved in lymphopoiesis.^49^ Furthermore, the aging-associated changes in human HSCs resulted in reduced proliferation, clonogenic potential, and attenuated productiton of lymphoid cells.^24,48^

The source of inflamm-aging is under investigation and dysbiosis has been identified as a major contributor to this phenomenon.^50–53^ In mice, lipopolysaccharide (LPS) from gut microbiota can accelerate inflammaging^54^; however, mice lacking the receptor for LPS (Toll-like receptor 4) have significantly lower levels of circulating pro-inflammatory cytokines.^55^ Cytokines play a key role in aging-associated increases in intestinal permeability, with TNF-*α* identified as a major driver of dysbiosis and compromised gut integrity.^53^ Additionally, in germ-free mice, the transfer of gut microbiota from aged, but not young, recipients was sufficient to induce systemic inflammation and increase the percentage of immunosuppressive T-regulatory cells (T-regs) in the spleen.^56^ Additionally, the accumulation of both visceral adipose tissue^57,58^ and senescent cells^59,60^ are thought to contribute to inflamm-aging. In addition to cell extrinsic factors promoting aging-associated immunosenescence, cell autonomous changes including increased DNA damage,^61^ mitochondrial dysfunction,^62^ and oxidative stress^63,64^ are thought to compromise the function of aged immune cells. These results suggest that treatments that reduce chronic inflammation, maintain microbial homeostasis, and mitigate mitochondrial dysfunction may improve hematopoiesis and immunity in aged hosts.

## 3 | IMMUNOTHERAPIES AND NEW HORIZONS FOR ELDERLY PATIENTS

### Definition of immunotherapy:

Immune-based therapies or immunotherapies are classes of treatments that prevent or target diseases with substances that regulate the immune system. Notable treatment options include recombinant cytokines (e.g., IFN-*α*, IL-2, and IL-12), antibodies that target immune checkpoints (e.g., PD-1, PD-L1, and CTLA-4), chimeric antigen receptor (CAR) T cells, bispecific T-cell engagers (BiTEs), and oncolytic viruses.

#### 3.1 | A brief history lesson on immunotherapies

The birth of immunotherapies originated from the field of bacteriology. Over 150 years ago, two German physicians, W. Busch and F. Fehleisen, documented that cancers significantly regressed in patients who accidentally developed erysipelas (a bacterial infection of the skin).^65,66^ W. Busch followed up this observation in 1868 by purposefully inducing erysipelas in cancer patients and again observed tumor shrinkage.^65,66^ In 1882, Fehleisen identified the causative agent of erysipelas as *Streptococcus pyogenes*.^65,66^ Despite these initial reports, the “Father of Immunotherapy” is widely considered to be an American surgeon named William Bradley Coley.^65,66^ In 1891, W.B. Coley treated bone and soft tissue sarcoma patients with Streptococcus.^65,66^ In his study, 66% of patients (n = 3) treated with live bacteria died, prompting him to administered heat-killed bacteria instead (which was also combined with heat-killed *Serratia marcescens*).^65,66^ This combinatorial approach was dubbed “Coley’s Toxin” and he reported high success rates in patients.^65,66^ However, his work was not highly appreciated or recognized at the time as it was considered anecdotal, poorly reproducible, and highly controversial.^65,66^

The results of Coley’s work would gain momentum in the 1960s, with the identification of T cells and their critical role in immunity being documented in 1967 by the French scientist Jacques Francis Albert Pierre Miller.^65,66^ The multitude of significant discoveries preceding and following the identification of T cells eventually resulted in FDA approval of immunotherapies to treat various solid tumors and hematological malignancies (Table 1).

The revolutionary potential of immunotherapies was recently recognized by the scientific community when Drs. James Allison and Tasuku Honjo were awarded the 2018 Nobel Prize for their groundbreaking work on checkpoint molecules as potential therapeutic targets. Based on the number of active clinical trials determining the efficacy of immunotherapies for the treatment of solid and hematological malignancies, what is clear is that the burgeoning field of immunotherapy will continue to expand and additional research will be needed to understand how immune-based drugs behave in various patient demographics, particularly those with compromised immune systems.

### 3.2 | Preclinical and clinical studies on aging and the efficacy of immunotherapies

Despite the increasing use of immunotherapies as treatments for patients with relapsed and refractory disease, and the growing number being tested as frontline options in clinical trials, preclinical studies using aged model systems to test the efficacy of immunotherapies and clinical trials enrolling aged patients are noticeably sparse.^9,13,67,68^

The scope of this section is to evaluate immunotherapy studies that used aged model systems and discuss results from clinical trials that reported AEs and treatment outcomes in aged study participants. Despite our desire to provide preclinical and clinical data summaries for each immunotherapy highlighted in Table 1, we were often hampered by the lack of reports containing one or both research modalities. Therefore, we have chosen to provide an assessment of select studies that delineate the impact of aging on the efficacy of immunotherapies in multiple disease settings. Furthermore, we attempted to summarize outcomes from clinical trials where age was independently evaluated, or the patient demographic had a median age of ≥65 years old.

#### 3.2.1 | *α*PD-1 antibody studies

The efficacy of immune checkpoint inhibitors (ICI) is partially dependent on the functional capacity of endogenous T-cells and the composition of the T-cell pool. In mice, the surface expression of PD-1 increases with age on CD4^+^ and CD8^+^ T cells resulting from a change in frequency of the T-cell repertoire from naïve to memory T cells.^69^ In addition to frequency changes, aged naïve CD4^+^ and CD8^+^ T cells exhibited significantly lower surface levels of CD127, CD25, and CD28 compared to naïve T cells isolated from young mice,^69^ which supports documented proliferative and survival defects in aged murine T cells.^24^ In addition to quantitative and qualitative changes in the aged T cells, similar alterations are observed in DCs. The frequency of CD8*α*^−^ DCs increases in the spleen, lung, and Peyer’s patches of aged mice, whereas a larger representation of myeloid DCs is found in the lymph node and lungs of aged relative to young mice.^69^ In addition to this aging-associated shift in DC frequency, aged DC subsets from multiple organs expressed higher levels of PD-L1 and PD-L2.^69^ In functional studies, the addition of *α*PD-1, *α*PD-L1, or *α*PD-L2 antibody treatment with *α*CD3 T-cell activating antibody did not rescue proliferative defects in aged CD4^+^ or CD8^+^ T cells.^69^ In addition to not rescuing proliferative defects, cytokine production (IFN-*γ*) was only modestly increased in aged T cells treated with *α*PD-1, *α*PD-L1, or *α*PD-L2 antibody and augmented function was mainly observed in aged T cells not expressing PD-1.^69^

Despite functional defects in aged T cells and higher surface PD-1 expression, a recent study demonstrates that the efficacy of *α*PD-1 antibody treatment increases in aged mice and patients with melanoma.^70^ Aged mice (≥10 months of age) transplanted with murine melanoma cells exhibited a significant decrease in tumor burden by 2 weeks posttreatment.^70^ Furthermore, there was a significant increase in the frequency of IFN-*γ* and TNF-*α* producing CD8^+^ T cells in tumor-bearing mice receiving *α*PD-1 antibody treatment compared to responses observed in young (6–10 weeks of age) mice.^70^ In addition to functional changes in effector T-cells, the ratio of CD8^+^ T-cells to T-regs was significantly higher in aged mice receiving *α*PD-1 antibody treatment for melanoma.^70^ The importance of T-regs in the response to *α*PD-1 antibody treatment was highlighted by the observation that depleting T-regs from young tumor-bearing mice receiving *α*PD-1 antibody treatment led to highest degree of suppression of tumor burden compared to single-agent treatment alone.^70^

In humans, *α*PD-1 antibody treatment for melanoma led to similar outcomes (progressive disease, stable disease, and complete responses) in young (<62 years of age; n = 238), older (≥62 and ≤75 years of age; n = 300), and elderly (>80 years old; n = 62) patients.^70,71^ The outcomes of these studies support similar trials of this scope enrolling older patients with melanoma (clinicaltrials.gov). Similar outcomes are observed in older patients receiving *α*PD-1 antibody treatment for NSCLC, where overall survival (OS) rates are equivalent in young and older patients (with a stratifying age of 70).^72^ Furthermore, toxicity profiles are similar between patients younger and older than 75 years of age.^73^

In all, these results suggest that despite the declining function and increased expression of PD-1 on aged T cells, the efficacy of *α*PD-1 antibody treatment is effective in older patients with various malignancies. Furthermore, the clinical studies suggest that safety profiles may not be impacted by age.

#### 3.2.2 | *α*CTLA-4 antibody studies

In murine studies, *α*CTLA-4 antibody treatment was ineffective at prolonging the survival of aged (>12 months old) mice challenged with triple negative breast cancer (TNBC) cells, whereas young (8–10 weeks) mice exhibited a significant extension in survival.^74^ The lack of protection with *α*CTLA-4 antibody treatment in aged mice was observed in two mouse strains (Balb/c and FVB) using two TNBC cell lines (4T1 and Met1).^74^ In clinical studies, seven trials have initiated (with two completed), which aim to determine the impact of Ipilimumab on TNBC outcomes. At this time, no results have been reported. These results suggest that the aged microenvironment compromises the efficacy of *α*CTLA-4 antibody treatment for TNBC. However, reports from clinical trials will be instrumental in determining the validity of this conclusion, which is currently hampered by limited preclinical and clinical studies.

In clinical studies of melanoma, 188 aged patients (>70 years of age) were evaluated for their tumor response at baseline, at the end of induction therapy, and throughout the 3-week treatment period for AEs, including those induced by immune cells.^75^ The responses in patients between 70 and 80 years of age (n = 118) and those ≤70 years of age (n = 645) with melanoma appeared equivalent after *α*CTLA-4 antibody treatment. Furthermore, immune-related best overall response rates, immune-related partial response rates, immune-related stable disease, immune-related progressive disease, and immune-related disease control rates profiles were similar between young and older patients.^75^ However, in patients >80 years of age (n = 26), the immune-related best overall response rate significantly declined.^75^ Despite changes in the response rates, which appeared to decline in the oldest cohort of patients in this study, the progression-free survival (PFS), OS, and treatment-related AEs were not statistically different between patients under and over 70 years of age receiving *α*CTLA-4 antibody treatment.^75^ Given the small number of patients over 80 years of age included in this study, PFS and OS were not determined for this population. Therefore, additional studies are needed to determine the safety and efficacy of *α*CTLA-4 antibody treatment in geriatric patients with melanoma.

Overall, these results demonstrate that the efficacy of *α*CTLA-4 antibody treatment in older patients (<80 years) with melanoma is comparable to younger patients; however, clinical results will be useful for ascertaining this information for older patients with TNBC. Regarding safety, treatment-related AEs in aged patients receiving *α*CTLA-4 antibody treatment for melanoma are relatively tolerable with an average of 60% of older patients (mean age of 59) reporting serious AEs in the clinical trials analyzed in this review (Table 2).

#### 3.2.3 | *α*CD40 and IL-2 studies

The impact of *α*CD40 and IL-2 combination treatment was determined in young (4 months) and old (22 months) mice.^76^ Unlike in young mice, old mice treated with high-dose combination therapy rapidly succumbed to a lethal cytokine release syndrome (CRS) characterized by high systemic levels of IL-6, IFN-*γ*, TNF-*α*, and severe gut, liver, and lung pathology.^76^ The multiorgan pathology observed in aged mice treated with immunotherapy was not impacted by the depletion of T or NK cells; however, the depletion of macrophages completely ameliorated the treatment-related toxicity observed in aged mice.^76^ Furthermore, depleting macrophages abrogated the lethal effects of high-dose *α*CD40 and IL-2 combination therapy in aged mice and this effect was also phenocopied with the TNF-*α* inhibitor etanercept.^76^ Of note, TNF-*α* secretion from LPS-stimulated human macrophages increased in an age-dependent manner with significant increases observed in cells from donors between 63 and 95 years of age relative to younger donors (28–59 years of age).^76^ In addition to protecting aged mice from the lethal effects of high-dose *α*CD40 and IL-2 treatment, the combination therapy of *α*CD40/IL-2/etanercept led to a significant extension in the survival of aged mice transplanted with Lewis lung carcinoma cells relative to those treated with *α*CD40/IL-2 or rIgG/PBS.^76^

In all, these data demonstrate that aging-associated changes in immune hemostasis can be lethal in aged mice treated with *α*CD40 and IL-2 combination immunotherapy. However, responses to this treatment regimen can be fined-tuned to reduce toxicity and optimize efficacy when macrophages are depleted or pro-inflammatory cytokines are directly targeted (etanercept). Recent studies have corroborated the therapeutic benefits of repolarizing^77^ or depleting macrophages^78^ as strategies to optimize *α*CD40 and IL-2 immunotherapy treatment in aged mice.

#### 3.2.4 | BiTE studies

The impact of age was assessed in two Phase 2 clinical trial reports determining the efficacy of single-agent blinatumomab (a BiTE which targets CD19 on B cells and CD3 on T cells) treatment in patients with relapsed/refractory B-precursor acute lymphoblastic leukemia (BP-ALL).^79^ In these studies, 225 patients were <65 years of age (median age = 34) and 36 patients were ≥65 years of age (median age = 70).^79^ After receiving two cycles of treatment, complete remission (CR) was achieved in 46% of younger patients and 56% of older patients,^79^ and survival differences did not differ between the two cohorts.^79^

Two clinically significant AEs known to occur with blinatumomab treatment are neurological events and CRS. Despite similar CR and OS rates, significantly more grade 3–4 neurological events occurred in older patients receiving blinatumomab treatment.^79^ Furthermore, the number of patients presenting with CRS was significantly higher in patients ≥65 years of age (10% for younger adults and 19% for older adults); however, no fatal treatment-related AEs were reported in this study.^79^

Results from this study demonstrate that blinatumomab treatment is effective in older patients (≥65 years of age) with relapsed/refractory BP-ALL; however, treatment-related AEs (particularly neurological complications and CRS) are more common in older patients.

### 3.3 | In the pipeline

In this section, we review clinical trial results reported in ClinicalTrials.gov, which specifically provide data on immunotherapy outcomes in aged patients. Of the 30 trials reviewed, only 11 reported data that was stratified by age group including those over 65 years of age (Table 3). Furthermore, many of the identified studies that fit our criteria were Phase I or Phase II clinical trials, highlighting the lack of clinical data available reporting on the impact of aging on the efficacy of immunotherapies. Therefore, we focused our analysis on the presentation of AEs in aged study participants. From this analysis, serious AEs (Grade 3 or 4) were reported in 45% of older patients treated with atezolizumab (n = 4 studies), 40% of older patients treated with ipilimumab (n = 4 studies), 36% of older patients treated with pembrolizumab (n = 2 studies), and 24% of older patients treated with sipuleucel-T (n = 1 study). Although limited for reasons stated above, our analysis suggests that close to 40% of older patients receiving ICI or sipuleuclel-T therapy will experience serious AEs during treatment, which is higher than toxicity rates observed in younger patients. Moving forward, it will be important to define efficacy and safety profiles for aged patients and to stratify the data by immunotherapy class and cancer type.

## 4 | CONCLUSIONS

### 4.1 | Current landscape

Immunotherapies have revolutionized the medical field by providing effective treatment options for patients with relapsed and refractory diseases such as melanoma, TNBC, NSCLC, prostate cancer, B-cell acute lymphoblastic leukemia, and diffuse large B-cell lymphoma.^80^ Furthermore, the development of novel immunotherapies continues to rise, and these classes of drugs are more frequently being tested in clinical trials as frontline therapies in combination with standard of care protocols.^80^

Despite numerous success stories, many patients will relapse within 3 years of treatment initiation, and in many cases, patients will fail to respond to frontline treatment.^81–84^ In those that do respond to treatment, serious AEs, including the onset of CRS, can reduce therapeutic efficacy due to the early termination of the treatment protocol.^85–89^ Given the staggering prices associated with immunotherapy treatment (≥$100,000-$500,000 in many cases^90,91^) and the potential of developing treatment-related toxicities, it is imperative to identify which patients will effectively respond to immunotherapy treatment.

#### 4.1.1 | Efficacy

There is a growing concern that altered immunological homeostasis will negatively impact the efficacy and safety of immunotherapy treatments in aged and elderly individuals.^92,93^ This belief stems from the well-established immunological decline documented in aged mice and humans.^24^ To this end, we sought to determine whether aging adversely impacts the efficacy of various classes of immunotherapy by reviewing studies conducted in aged murine models and clinical trials enrolling aged study participants. In our assessment of preclinical literature and reviews of clinical trial data, it appears that aging does not significantly impair the efficacy *α*PD-1 and *α*CTLA-4 immunotherapy in murine models and in patients <80 years of age. In fact, it appears that older patients respond better to ICI therapies compared to younger patients. The age-dependent response differences may reflect aging-associated increases in PD-1 and CTLA-4 surface expression on T cells,^69,94–96^ thus the “release” from immune suppression in aged patients receiving ICI therapy may be more apparent than responses observed in young patients.

In addition to ICI therapy, the efficacy of blinatumomab was similar between young (a mean of 34 years of age; n = 225) and aged (a mean of 70 years of age; n = 36) patients with relapse/refractory BP-ALL.^79^ These observations suggest that aging-associated declines in T-cell function do not limit the efficacy of blinatumomab, *α*PD-1 antibody therapy, and *α*CTLA-4 antibody therapy in patients <80 years old. However, in patients >80 years old receiving *α*CTLA-4 antibody therapy (n = 26^75^) or *α*PD-1 antibody therapy (n = 62^71^) to treat melanoma, overall response rates and OS decline. These results suggest that in very old patients receiving ICI therapy, the loss of immune homeostasis maybe a major barrier to treatment success. Overall, these findings suggest that the efficacy of immunotherapies may differ in patients less than and older than 80 years of age. Before definitive conclusions can be made, future studies should include larger samples sizes (particularly those >80 years of age) and testing should be performed on more classes of immunotherapies.

#### 4.1.2 | Safety

Serious AEs were more frequently reported in aged mice receiving immunotherapy treatment. In preclinical models, high-dose *α*CD40/IL-2 combination treatment was lethal in aged but not young mice,^76^ which was mitigated by the TNF-*α* inhibitor etanercept. Furthermore, in the studies reviewed, a significant portion of aged patients developed serious AEs when receiving immunotherapy treatment, which is supported by results from ongoing clinical trials (Table 3). These observations may reflect the impact of deregulated inflammation in aged mice and humans, which may predispose older patients to serious AEs, including the manifestation of CRS after receiving immunotherapy treatment.

### 4.2 | Moving forward

The usage of immunotherapies as treatments for cancer is still in its infancy. Refinements in the design of preclinical and clinical trials are necessary to determine how aging impacts the efficacy and safety of each class of immunotherapy. Specific areas of improvements should be considered moving forward.

#### 4.2.1 | Preclinical models

- Increasing the number of immunotherapy studies conducted in aged model systems.^97^
- Using humanized mice (reconstituted with aged hematopoietic and immune cells) to determine efficacy and safety profiles for various classes of immunotherapies and cancer types.
- Standardizing what is considered “middle-aged” and “old” in murine studies. Given emerging reports that the efficacy of certain classes of immunotherapies may decline in patients >80 years of age, stratifying murine-based studies into “middle-aged; 10–14 months of age” and “old; >18 months of age” may be useful in determining the impact of advanced age on immunotherapy efficacy and safety.^98–100^

#### 4.2.2 | Clinical trials

- Recruit and enroll more patients ≥65 years of age in immunotherapy-focused clinical trials: In the United States, people ≥65 years of age account for 61% of all new cancer cases and 70% of all cancer deaths; however, their enrollment in oncology-focused clinical trials active between 1993 and 1996 was only 25%.^101^
- Report age-group specific information on AEs and survival outcomes: As reported in this review and stated by others, specific data regarding AEs and outcomes (PFS and OS) are not routinely reported for older patients enrolled in clinical trials. The ability to glean these data from current and future clinical trials will be critical in establishing safety and efficacy profiles for specific classes of immunotherapies in aged patients.
- Change federal laws to require ≥50% of study participants enrolling in oncology-focused clinical trials to be ≥65 years of age when the immunotherapy being tested targets aging-associated diseases. Federal laws require that cancer trials enroll representative samples of women and members of underrepresented groups; however, these laws do not exist for elderly patients. A review of elderly patients’ enrollment in cancer drug registrations by U.S. Food and Drug Administration (USFDA) found statistically significant underrepresentation of the elderly study participants.^102^

### 4.3 | New horizons

Immunotherapies are showing significant clinical promise in older patients with intractable diseases in the few studies conducted to date in this patient demographic. We are truly living in exciting times where older patients have more therapeutic opportunities to explore than ever before. Due to the tremendous success documented in young patients, and emerging data demonstrating efficacy in older patients, novel immune-based therapies are being generated and tested at a fast pace that will further increase treatment options moving forward.^103,104^

**FIGURE 2:**
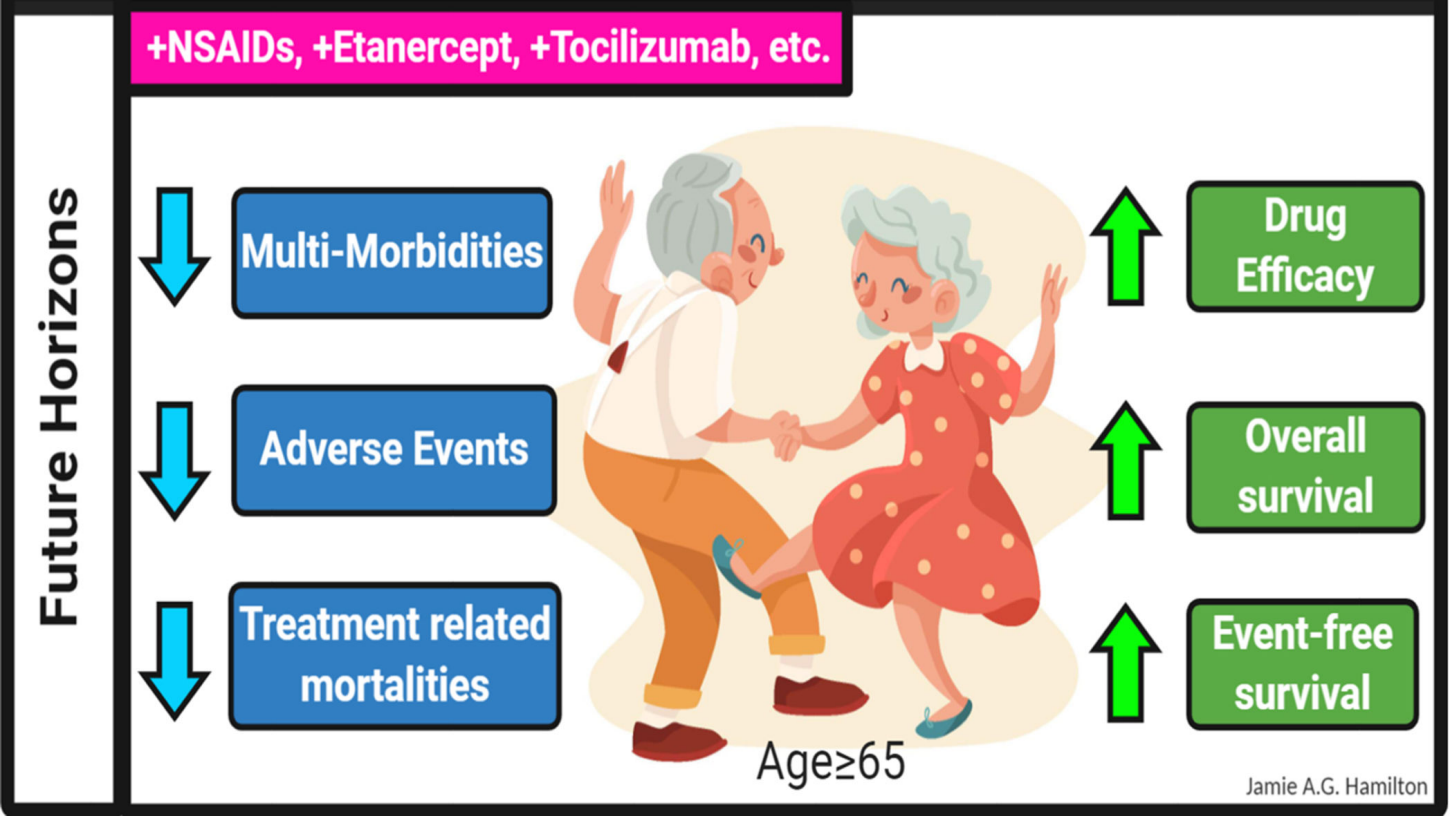
Combining anti-inflammatory agents with immunotherapies represents an attractive strategy that should be explored in future preclinical and clinical settings

As highlighted at several points in this review, serious AEs appear to be a common manifestation in aged patients receiving immunotherapies; however, the frequency and severity of these events may be disease and treatment specific. In pediatric patients receiving CAR T-cell treatment for relapsed and refractory B-cell malignancies, CRS is a common serious AE, which is successfully mitigated by treatment with the IL-6 inhibitor tocilizumab.^89,105,106^ Given this observation, the inclusion of anti-inflammatory drugs in immunotherapy treatment backbones should also be explored as a therapeutic option for treating CRS in aged patients. Aging in mice and humans is accompanied by the onset of chronic inflammation (also referred to as “inflamm-aging^107^”), and the impact of this state on the safety and efficacy of immunotherapies in aged patients should be considered in future studies. Given the multitude of anti-inflammatory drugs that are currently approved for use in humans (Table 4), rational combinatorial treatment strategies could be easily explored in preclinical models and clinical settings.^108–111^ Targeting deregulated inflammation represents an attractive approach with documented preclinical and clinical success, and thus, the future of immunotherapy treatment in aged populations looks even brighter (Figure 2).

## Figures and Tables

**TABLE 1:** A brief history of immunotherapies

Immunotherapy	Year approved	Target cancer(s)
Interleukin-2 (IL-2)	1991	Metastatic kidney cancer
Rituximab (CD20 targeting monoclonal antibody)	1997	B-cell leukemia and lymphoma
Interleukin-2 (IL-2)	1998	Metastatic melanoma
Sipuleucel-T (activated autologous PBMCs combined with recombinant fusion protein PA2024)	2010	Castration-resistant prostate cancer
Ipilimumab (CTLA-4 targeting monoclonal antibody)	2011	Melanoma
Blinatumomab (bispecific T-cell engager [BiTE] targeting CD19 on B cells and CD3 on T cells)	2014	B-cell precursor acute lymphoblastic leukemia
Nivolumab (PD-1 targeting monoclonal antibody)	2014, 2015	Melanoma, non-small cell lung cancer
Talimogene laherparepvec (T-VEC), (first oncolytic virus)	2015	Metastatic melanoma
Elotuzmab (SLAMF7-targeting monoclonal antibody)	2015	Multiple myeloma
Pembrolizumab (PD-1 targeting monoclonal antibody)	2017	Urothelial cancer
Axicabtagene ciloleucel (CD19-directed CAR T cells)	2017	Large B-cell lymphoma
Gemtuzumab ozogamicin	2017	Acute myeloid leukemia
Tisagenlecleucel (CD19-directed CAR T-cells)	2017, 2018	B-cell acute lymphoblastic leukemia, diffuse large B-cell lymphoma
Atezolizumab (PD-L1 targeting monoclonal antibody)	2017, 2019	Urothelial cancer, triple-negative breast cancer
Nivolumab + ipilimumab + chemotherapy	2020	Metastatic non-small cell lung cancer
Gemtuzumab ozogamicin	2020	Acute myeloid leukemia

* This list is not exhaustive.

**TABLE 2:** A list of selective clinical trials registered in ClinicalTrials.gov that included ipilimumab treatment for melanoma

Drug	Clinical trial-official title	PMID	Study id	Cancer/trial phase	Patients age Mean/ Median = X (range or SD)	Total No. of patients	Serious AE: affected/total at risk (%)
Ipilimumab	Addition of ipilimumab (MDX-010) to isolated limb infusion (ILI) with standard melphalan and dactinomycin in the treatment of advanced unresectable melanoma of the extremity	—	NCT01323517	Mel/Phase I	Median = 64 (37–80)	26	10/26 (38.46%)
Ipilimumab	A Phase Ib study of Yervoy with Sylatron for patients with unresectable stages IIIB/C/IV melanoma	28031816	NCT01496807	Mel/Phase I	Median = 65 (38–83)	31	14/31 (45.16%)
Ipilimumab	Pilot ipilimumab in Stage IV melanoma receiving palliative radiation therapy	27681753	NCT01449279	Mel/Phase II	Mean = 59 (18–89)	22	11/22 (50.00%)
Ipilimumab	Phase 2 study of ipilimumab plus dacarbazine in Japanese patients with advanced melanoma	26407818	NCT01681212	Mel/Phase II	Mean = 55 (12.66)	15	14/15 (93.33%)
Ipilimumab	Phase 2 study of ipilimumab in Japanese advanced melanoma patients	26410424	NCT01990859	Mel/Phase II	Median = 62.5 (29–76)	20	11/20 (55.00%)
Ipilimumab	Study of nivolumab given sequentially with ipilimumab in subjects with advanced or metastatic melanoma (CheckMate 064)	27269740	NCT01783938	Mel/Phase II	Mean = 59.8 (14.61)	138	51/70 (72.86%)^b^
Ipilimumab	Phase 3 trial in subjects with metastatic melanoma comparing 3 mg/kg ipilimumab versus 10 mg/kg ipilimumab	28359784	NCT01515189	Mel/Phase III	Mean = 59.7 (13.92)	726	3 mg/kg, 194/362 (53.59%) 10 mg/kg, 245/364 (67.31%)
Ipilimumab	a comparative study in Chinese subjects with chemotherapy naïve stage IV melanoma receiving ipilimumab (3 mg/kg) versus dacarbazine	—	NCT02545075	Mel/Phase III	Mean = 53.8 (13.56)	182	34/122 (27.87%)^a^
Ipilimumab	Efficacy study of ipilimumab versus placebo to prevent recurrence after complete resection of high-risk Stage III melanoma	25840693	NCT00636168	Mel/Phase III	Mean = 51.1 (12.86)	951	257/471 (54.56%)^c^
Ipilimumab	Ipilimumab + temozolomide in metastatic melanoma	—	NCT01119508	Mel/Phase II	Median = 62 (33–75)	64	59/64 (92.19%)

a Ipilimumab arm of trial.

b Ipilimumab followed by nivolumab.

c Ipilimumab arm of trial.

**TABLE 3:** A list of selective clinical trials registered in ClinicalTrials.gov that enrolled and provided data specifically on aged (>65) patients with cancer

Drug	Clinical trial-official title	PMID	Study id	Cancer/tria phase	Patients age Mean/ Median = X (range or SD)	(Review again) Total No. of patients	Serious AE: affected/total at risk (%)
Atezolizumab	A study of atezolizumab compared with chemotherapy in participants with locally advanced or metastatic urothelial bladder cancer [IMvigor211]	29268948	NCT02302807 (2014–2019)	BLC/ Phase III	Mean = 65.9	459	192/459 (41.83%)^a^
Atezolizumab	A study of atezolizumab as first-line monotherapy for advanced or metastatic non-small cell lung cancer (B-F1RST)	—	NCT02848651 (2016–2020)	NSCLC/ Phase II	Mean = 68.7	152	81/152 (52.29%)
Atezolizumab	A study of atezolizumab in participants with programmed death-ligand 1 (PD-L1) positive locally advanced or metastatic non-small cell lung cancer (BIRCH)	28609226	NCT02031458 (2014–2020)	NSCLC/ Phase II	Median = 66.8	138	47/138 (34.06%)^b^
Atezolizumab	A study of atezolizumab in participants with programmed death-ligand 1 (PD-L1) positive locally advanced or metastatic non-small cell lung cancer (NSCLC) [FIR]	29775807	NCT01846416 (2013–2019)	NSCLC/ Phase II	Mean = 65.7	137	69/137 (50.36)^c^
Ipilimumab	A phase Ib study of Yervoy with sylatron for patients with unresectable stages IIIB/C/IV Melanoma	28031816	NCT01496807 (2011–2017)	Mel/Phase I	Median = 65	31	14/31 (45.16%)
Ipilimumab	Evaluation of circulating T cells and tumor infiltrating lymphocytes with specificities against tumor associated antigens during and after neoadjuvant chemotherapy and phased ipilimumab in non-small cell lung cancer	29258674	NCT01820754 (2013–2018)	NSCLC/ Phase II	Mean = 65.3	24	6/24 (25.00%)
Ipilimumab	A phase 2, randomized, double-blind study of ipilimumab administered at 3 mg/kg versus 10 mg/kg in adult subjects with metastatic chemotherapy-naïve castration resistant prostate cancer who are asymptomatic or minimally symptomatic	—	NCT02279862 (2014–2018)	PC/ Phase II	Mean = 66	51	21/51 (41.17%)^c^
Ipilimumab	Phase II study of combined ionizing radiation and ipilimumab in metastatic non-small cell lung cancer (NSCLC)	30397353	NCT02221739 (2014–2020)	NSCLC/ Phase II	Median = 68	39	19/39 (43.59%)
Ipilimumab	A phase I/II, open-label, dose-escalation study of MDX-010 administered every 3 weeks for four doses in patients with metastatic hormone-refractory prostate cancer	23535954	NCT00323882 (2006–2014)	PC/ Phase I & II	Mean = 65.7	71	35/71 (49.29%)^c^
Pembrolizumab	Randomized phase 2 trial of ACP-196 and pembrolizumab immunotherapy dual CHECKpoint inhibition in platinum resistant metastatic urothelial carcinoma (RAPID CHECK study)	—	NCT02351739 (2015–2019)	UC/ Phase II	Mean = 65.8	35	15/35 (42.86%)^d^
Pembrolizumab	Phase 2B single-site, open-label, nonrandomized study evaluating the efficacy of neoadjuvant pembrolizumab for unresectable stage III and unresectable stage IV melanoma	—	NCT02306850 (2014–2019)	MEL/ Phase II	Mean = 66.3	10	3/10 (30.00%)
**Sipuleucel-T**	A randomized, double blind, placebo controlled phase 3 trial of immunotherapy with autologous antigen presenting cells loading with PA2024 (Provenge(R), APC8015) in men with metastatic androgen independent prostatic adenocarcinoma d treatment. y. lotherapy, arm 1 of study. study.	20818862	NCT00065442 (2003–2010)	PC/ Phase III	Median = 71.1	341	82/338 (24.26%)^e^

a Patients who received treatment.

b Cohort-1 in this study.

c All cohorts in study.

d Pembrolizumab monotherapy, arm 1 of study.

e Sipuleucel-T arm of study.

**TABLE 4:** Anti-inflammatory drugs which may improve the efficacy and safety of immunotherapies in aged patients with cancer

Drug	Current understanding of MOA	Used in patients ≥65 years old	AGS 2019 BEERS recommendation (PMID: 30693946)	Cancer-specific clinical trial ID, patients ≥65 years old
Diclofenac	Non-selective COX-1/COX-2 inhibition	Yes	Avoid chronic use	NCT00601640
Diflunisal	Prostaglandin-synthase inhibitor	Yes	Avoid chronic use	—
Etanercept	Tumor necrosis factor (TNF) inhibitor	Yes	N/A	NCT00201838
Etodolac	COX-2 preferential inhibitor, can inhibit COX-1	Yes	Avoid chronic use	NCT00527319
Fenoprofen	Prostaglandin-synthase inhibitor	Yes	Avoid chronic use	—
Flurbiprofen	Non-selective COX-1/COX-2 inhibition	Yes	Avoid chronic use	—
Ibuprofen	Non-selective COX-1/COX-2 inhibition	Yes	Avoid chronic use	—
Indomethacin	Non-selective COX-1/COX-2 inhibition	No	Avoid use	NCT00210470
Ketoprofen	COX-2 preferential, but can inhibit COX-1, may inhibit bradykinin	Yes	Avoid chronic use	—
Ketorolac	Non-selective COX-1/COX-2 inhibition	Yes	Avoid use	NCT01806259
Mefenamic acid	Non-selective COX-1/COX-2 inhibition	Yes	Avoid chronic use	—
Meloxicam	COX-2 preferential, can inhibit COX-1	Yes	Avoid chronic use	—
Nabumetone	COX-2 preferential, can inhibit COX-1	Yes	Avoid chronic use	—
Naproxen	Non-selective COX-1/COX-2 inhibition	Yes	Avoid chronic use	NCT01712009
Oxaprozin	Non-selective COX-1/COX-2 inhibition	Yes	Avoid chronic use	—
Piroxicam	COX-1 inhibitor	Yes	Avoid chronic use	—
Sulindac	Non-selective COX-1/COX-2 inhibition	Yes	Avoid chronic use	NCT00368927
Tocilizumab	Inhibits the binding of IL-6 to the IL-6R	Yes	N/A	NCT00883753
Tolmetin	Prostaglandin-synthase inhibitor	Yes	Avoid chronic use	—
